# Comprehensive Proteomic Analysis of Common Bean (*Phaseolus vulgaris* L.) Seeds Reveal Shared and Unique Proteins Involved in Terminal Drought Stress Response in Tolerant and Sensitive Genotypes

**DOI:** 10.3390/biom14010109

**Published:** 2024-01-15

**Authors:** Mayavan Subramani, Carlos A. Urrea, Sowjanya R. Tamatamu, Venkateswara R. Sripathi, Krystal Williams, Lathadevi K. Chintapenta, Antonette Todd, Gulnihal Ozbay

**Affiliations:** 1 Molecular Genetics and Epigenomics Laboratory, College of Agriculture, Science and Technology (CAST), Delaware State University, Dover, DE 19901, USA; krystald.williams5@gmail.com (K.W.); atodd@desu.edu (A.T.); 2 Panhandle Research Extension and Education Center, University of Nebraska, 4502 Avenue I, Scottsbluff, NE 69361, USA; currea2@unl.edu; 3 Center for Molecular Biology, Alabama A&M University, Normal, AL 35762, USA; sowjanyatamatamu@gmail.com (S.R.T.); v.sripathi@aamu.edu (V.R.S.); 4 Biology Department, College of Arts and Sciences (CAS), University of Wisconsin-River Falls, River Falls, WI 54022, USA; lathadevi.chintapenta@uwrf.edu; 5 Department of Agriculture and Natural Resources, Delaware State University, 1200 North DuPont Highway, Dover, DE 19901, USA

**Keywords:** proteomics, terminal drought stress, common bean, genotypes, differentially expressed proteins

## Abstract

This study identified proteomic changes in the seeds of two tolerant (SB-DT3 and SB-DT2) and two sensitive (Merlot and Stampede) common bean genotypes in response to terminal drought stress. Differentially expressed proteins (DEPs) were abundant in the susceptible genotype compared to the tolerant line. DEPs associated with starch biosynthesis, protein–chromophore linkage, and photosynthesis were identified in both genotypes, while a few DEPs and enriched biological pathways exhibited genotype-specific differences. The tolerant genotypes uniquely showed DEPs related to sugar metabolism and plant signaling, while the sensitive genotypes displayed more DEPs involved in plant–pathogen interaction, proteasome function, and carbohydrate metabolism. DEPs linked with chaperone and signal transduction were significantly altered between both genotypes. In summary, our proteomic analysis revealed both conserved and genotype-specific DEPs that could be used as targets in selective breeding and developing drought-tolerant common bean genotypes.

## 1. Introduction

Common bean (*Phaseolus vulgaris* L.) is a protein-rich grain legume that contributes to the world’s food security and nutrition. Different classes of common beans have different seed sizes, colors, and shapes [1]. The yield of common beans drops by 10 to 100% when exposed to terminal and intermittent drought stress; in particular, the seed weights drop by 25% [2]. Drought stress reduces the photosynthetic rate, thus diminishing the assimilated export to sink organs such as seeds, fruits, flowers, and roots. As a result, a reduction in starch and protein accumulation affects the seed sink strength [3]. Weather conditions and genotypes also influence seed protein content during drought [4].

Studying the mechanism of drought stress tolerance at the molecular level can aid in advancing the crop development program [5]. Like other crops, identifying the molecular mechanisms underlying drought stress response, susceptibility, and tolerance in common beans remained challenging [6]. Slow progress in identifying the genes responsible for drought tolerance in common beans has been achieved in the last decade due to the availability of the whole genome and transcriptome sequences [7,8,9,10]. However, understanding the biological function of specific stress-responsive genes is still unclear. It is possible to efficiently elucidate the molecular mechanisms behind drought stress responses and study the functional analysis of translated genomic regions using modern proteomic approaches [11]. Further, identifying and validating the drought-responsive proteins hold a promising approach in marker-assisted breeding in the future [12].

Proteomics studies related to drought stress in legumes have been primarily reported in soybean (*Glycine max* L.) [13], cowpea (*Vigna unguiculata*) [14], chickpea (*Cicer arietinum*) [15], mung bean (*Vigna radiata*), and peanut (*Arachis hypogaea* L.) [16]. However, only some proteomics studies have been reported in common beans, emphasizing biotic and abiotic stresses [17]. Most proteomics studies on legumes under abiotic stress focused on leaves, shoots, and roots [18]. This study aimed at profiling differentially expressed proteins (DEPs) from seeds among four diverse genotypes. Investigating DEPs from all organs at various developmental stages, including shoots, roots, leaves, flowers, pods, and seeds, may reveal novel pathways crucial for improving legumes under stressed or unstressed conditions [19]. The correlation between developing seed traits and phenotypic markers in drought stress has been reported [20,21]. The effect of drought stress on the amino acid and nutrient content of the seeds due to transportation has been reported [2]. Studies have demonstrated that tolerant genotypes of plants maintain a better leaf water status, facilitating sucrose transport into seeds during development [22,23]. Seed protein quality and content are influenced by genotype and drought stress [24]. The seed protein content of tolerant and sensitive genotypes under drought and non-stress conditions has been reported [24,25].

However, the molecular mechanisms involved in the interaction between terminal drought stress and seed proteins still need to be fully understood. In the present study, we investigated four genotypes, two tolerant (SB-DT2 and SB-DT3) and two sensitive genotypes (Merlot and Stampede) of common beans in response to terminal drought stress. These genotypes are derived from the Mesoamerican and Durango gene pools and belong to the Mesoamerican and Durango races, respectively. They represent the leading market class in the United States (NASS, 2020). Using transcriptomics (RNA-Seq) and metabolomics approaches, we recently reported that the tolerant genotypes SB-DT2 and SB-DT3 perform well even under drought stress conditions [26,27]. In contrast, drought stress conditions have significantly reduced the yield and performance of Merlot and Stampede (sensitive) genotypes under drought stress conditions [28]. We hypothesize that the accumulation of differentially expressed proteins (DEPs) specific to each genotype, along with the biological pathways and processes associated with those DEPs, is influenced by the genetic background and developmental phase of each genotype during terminal drought stress. These genotype and development-dependent differences in DEP profiles may reveal key regulatory processes and adaptation mechanisms that confer improved performance under terminal drought conditions.

We conducted a gel-free proteomic analysis of four common bean genotypes (susceptible and tolerant) under irrigation and terminal drought stress conditions. Further, we investigated the associated pathways and biological processes of DEPs in four genotypes to better understand the underlying molecular mechanisms in response to drought stress.

The primary objective of our study was to profile the seed proteomics of both tolerant and sensitive genotypes in response to terminal drought stress. Our focus lies in analyzing the proteomics of seeds under terminal drought stress conditions to comprehend the changes in proteomic profiles in tolerant and sensitive genotypes. We aim to uncover variations in pathways and biological processes that occurred in these genotypes. This approach will enable us to identify potential differentially expressed proteins (DEPs) and understand the associated pathways and biological processes in both sensitive and tolerant genotypes. Previous studies, by [29,30] have elucidated changes in nitrogen content leading to modifications in the quality and quantity of proteins. These studies also highlighted variations in the concentration of specific proteins in seeds under drought-stress conditions. We are keen on delving into the seed proteomics of tolerant and sensitive genotypes in response to terminal drought stress and exploring the related changes in pathways and biological processes in these two genotypes.

## 2. Materials and Methods

### 2.1. Plant Materials and Experiments

Four common bean genotypes (Supplementary Table S1), two tolerant (SB-DT3 and SB-DT2), and two sensitive (Merlot and Stampede) to drought, were grown in Scottsbluff, NE research station with a silty-loam soil of 75% silt, 15% sand, and 10% clay located at an elevation of 1240 m, and with a latitude of 41°56.6′ N and a longitude of 103°41.9′ W. The soil health and the history of agricultural practices followed were available for the site. A detailed experimental design has been reported [26,28]. This study utilized a randomized block design (RBD) with four replications. The plots selected at the site have four 3.6 m crop rows with 56 cm spacing. Terminal drought stress was induced when at least 50% of the plants in each row in the plot reached the flowering stage, and no irrigation was provided to the plants post anthesis. Plants were randomly harvested at the R7 stage of bean development from the middle rows of each plot after 2–3 weeks of drought stress. A total of 24 common bean seed proteome samples were collected from two sensitive and two tolerant genotypes with three replicates each. The genotypes and their respective races were specified in [26]. Supplementary Table S1 details the physiological performances, including yield, flowering, maturity days, and 100 seed weight, under both stress and control conditions. An overview of data interpretation is presented in Supplementary Figure S1.

### 2.2. Sample Preparation and Digestion

The total protein from seeds was extracted based on [31] with modifications. Approximately 100–200 mg of seed tissue were ground into a powder with liquid nitrogen. The powder was mixed with lysis buffer (100 mM Tris-HCl, pH 8.8, 2% SDS, 2% 2-mercaptoethanol, 5 mM EGTA, 10 mM EDTA, 1 mM PMSF, 1% plant protease inhibitor cocktail) (Sigma-Aldrich Co., St. Louis, MO, USA, Cat.No. P9599-5ML ), and the supernatant was collected following centrifugation at 14,000 rpm for 30 min at 4 °C. The protein concentrations were assessed using bicinchoninic acid (BCA).

Sample preparation and analysis were based on [32]. Briefly, 100 mL of sample was mixed with 200 mL of 50 mM ammonium bicarbonate. The mixture was incubated at 56 °C for 1 h with 10 mM dithiothreitol (DTT). The sample mixture was alkylated in the dark using 20 mM IAA (iodoacetamide). After washing with 50 mM ammonium bicarbonate, free trypsin was added in a ratio of 1:50 and incubated overnight at 37 °C.

### 2.3. Nano LC-MS/MS Analysis

One microgram of enriched peptide was dissolved in mobile phase A containing 0.1% formic acid. The elution gradient was increased from 2 to 8% in mobile phase B (0.1% formic acid in 80% acetonitrile) over 3 min, 8% to 20% in 56 min, 20% to 40% in 37 min, and then 40% to 90% for the final 4 min. All the liquid chromatography–tandem mass spectrometry (LC-MS/MS) was conducted on the Ultimate 3000 nano UHPLC system (Thermofisher Scientific, Inc., Waltham, MA, USA) with a flow rate of 250 nL/minute with trapping column (PepMap C18, 100 Å, 100 mm × 2 cm, 5 mm) and an analytical column (PepMap C18, 100 Å, 75 mm × 50 cm, 2 mm). Peptides were selected for MS/MS analysis. The fragments were detected at a resolution of 15,000 with a fixed mass of 200 *m*/*z*. The automatic gain control (AGC) was set to 1 × 10^5^ The MS data were analyzed against the *Phaseolus vulgaris* L. (Kidney bean) (French bean) protein database at the UniProt (https://www.ebi.ac.uk, accessed on 27 March 2022)) using Maxquant (1.6.2.14). Trypsin was specified as a cleavage enzyme, with the maximum missed cleavage set to 2. The mass tolerance for precursor ions was set to 10 ppm, with the MS/MS tolerance of 0.6 Da. Only highly confident peptides were selected for the downstream protein analysis. Fold changes were calculated as drought/control.

### 2.4. Functional Enrichment Analysis

Gene ontology (GO) annotations for the common bean proteome were derived from the UniProt-GOA database (http://www.ebi.ac.uk/GOA/, accessed on 27 March 2022). Protein IDs that were identified were transformed into UniProt IDs and subsequently annotated with Gene Ontology (GO) IDs. Based on GO annotations, proteins were divided into three categories: biological process, cellular compartment, and molecular function. Fisher’s exact test was employed to assess the significantly modulated proteins. Proteins of relative quantitation were divided into two categories: fold change (FC) > 1.2 was considered upregulation, and FC < 0.83 was considered downregulation. The GO with a corrected *p*-value < 0.05 was considered significant. The Clusters of Orthologous Groups (KOGs) for eukaryotes were also used to classify the proteins.

The metabolic pathways were assigned to significant (*p*-value < 0.05) differentially expressed proteins (DEPs) using the Kyoto Encyclopedia of Genes and Genomes (KEGG) database. A summary of the software and databases used in the study is presented (Supplementary Table S2).

### 2.5. Quantitative PCR

To validate the proteomic results, genes corresponding to the identified DEPs were selected for quantitative real-time PCR (qRT-PCR) analysis. Primers used for the selected genes are provided in Supplementary Table S3. Relative gene expression was determined by the comparative CT (ΔΔCT) method [33]. PCR reactions were performed following the protocol outlined in [27].

## 3. Results

### 3.1. Proteomic Profiling of Genotypes in Response to Stress

**Figure 1:**
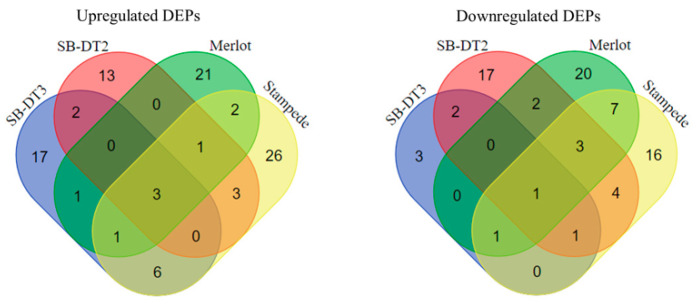
Using a Venn diagram to visually represent the unique and common upregulated and downregulated DEPs in both the tolerant and sensitive genotypes.

This study compared the proteome profiles of two drought-tolerant (SB-DT2 and SB-DT3) and two drought-sensitive (Merlot and Stampede) common bean genotypes to identify key proteins and pathways associated with drought tolerance. The proteome profiles of diverse or contrasting genotypes vary due to differences in storage protein content and germination rate. However, the storage protein content and germination rate are inversely proportional, changing with the developmental phases [34]. Specifically, tolerant plants adapt to stress conditions by developing unique anatomical structures or by employing physiological and molecular mechanisms. Cell wall-specific proteins or enzymes, membrane-linked proteins, transporters, transcription factors, R-proteins, and signaling molecules are often associated with these mechanisms and unique structures. This study identified 361 DEPs, and these DEPs were categorized into upregulated (Log2FC ≥ 1.2) and downregulated (Log2FC ≤ 1/1.2) based on fold change with significance (*p*-value < 0.05). Different stress conditions (drought/control) were compared within each genotype, and the resulting lists of DEPs for each genotype were then compared to those of other genotypes. Among the 361 DEPs, the upregulated and downregulated proteins identified in SB-DT3, SB-DT2, Merlot, and Stampede were 39 and 12, 46 and 47, 42 and 56, and 59 and 60, respectively (Supplementary Table S4). Seven and 11 DEPs were commonly identified between tolerant and sensitive genotypes, respectively (Table 1). The results obtained from the Venn diagram analysis (Figure 1) indicate that there was a low presence of common DEPs among the genotypes. On the other hand, most DEPs were observed to be unique to each genotype. Furthermore, the sensitive genotypes, Stampede and Merlot, exhibited a higher number of uniquely upregulated and downregulated DEPs, respectively, in comparison to the other genotypes.

### 3.2. Functional Analysis of Stress-Responsive Proteins

#### 3.2.1. Gene Ontology Annotations

**Figure 2:**
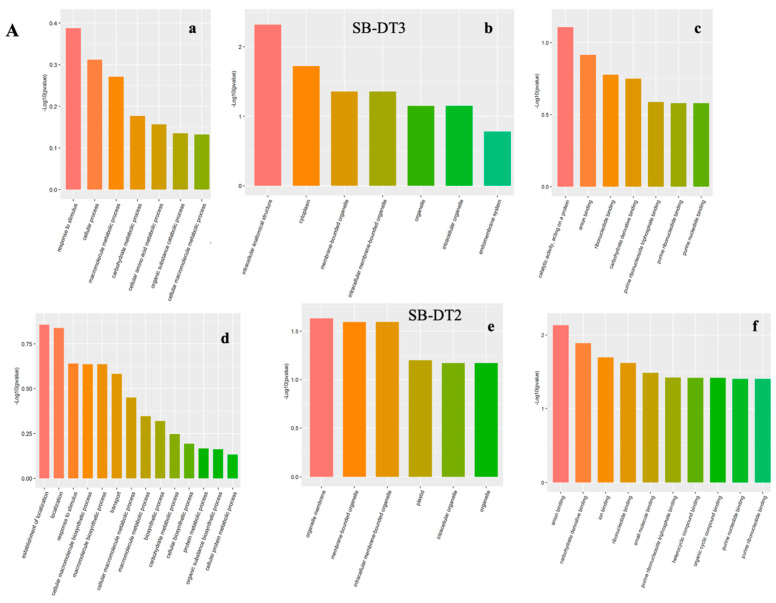

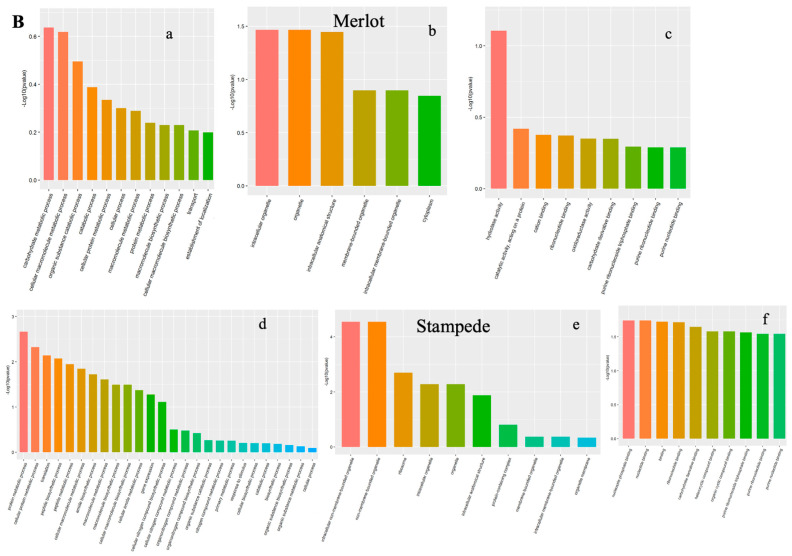
Gene ontology (GO) annotation of DEPs identified in four genotypes (**A**,**B**) under terminal drought stress conditions. The identified proteins were categorized into biological process (**a**,**d**), cellular component (**b**,**e**) and molecular function (**c**,**f**), GO domains. The analysis reveals both common and genotype—specific functional profiles of the proteomes during drought stress.

DEPs in the four genotypes were first enriched based on the GO database and categorized into biological processes (BP), cellular components (CC), and molecular functions (MF) using GO terms. As compared to the CC and MF classes, a significant number of BP categories were unique to the tolerant and sensitive genotypes in response to terminal drought stress (Figure 2, Supplementary Table S5).

#### 3.2.2. Biological Process (BP)

The significantly enriched GO BP classes and associated DEPs were further classified as conserved and unique proteins identified in each genotype. Among the top BP categories identified in SB-DT2, SB-DT3, Merlot, and Stampede drought tolerance were a response to stimulus/signaling, the establishment of localization, carbohydrate metabolism, and protein metabolism, respectively.

#### 3.2.3. Cellular Components (CC)

Similarly, the top enriched GO CC classes identified in SB-DT2, SB-DT3, Merlot, and Stampede genotypes were intracellular anatomical structure, organelle membrane, and intracellular membrane and non-membrane bounded organelles. Among the significant DEPs associated with CC categories, non-membrane-bound organelles were identified as unique proteins in the Stampede genotype when stressed and unstressed proteomes were compared.

#### 3.2.4. Molecular Function (MF)

The significant functional classes associated with DEPs identified in all four genotype comparisons were related to purine ribonucleotide triphosphate binding, ion binding, cation binding, and nucleotide phosphate binding.

### 3.3. Elucidating Drought Stress-Altered Biological Processes

This study is focused on deciphering the pathways affected by drought stress. A comprehensive enrichment assessment focusing on BP was conducted using a directed acyclic graph (DAG) analysis. The significant BP classes identified in four genotypes in response to terminal drought stress include starch biosynthesis, protein–chromophore linkage, and photosynthesis. Nonetheless, a few distinctive genotype-specific BP classes were identified. The specific BP classes identified in SB-DT3 were responses to metal/cadmium ion and glutamine family amino acid metabolism. Similarly, SB-DT2 showed exclusivity in response to abscisic acid and to biotic stimulus. In Merlot, the BP classes identified were macromolecule catabolism, energy reserve metabolism, and protein catabolism. In comparison, BP categories found in Stampede include response to oxygen-containing compounds, water, acid chemical stimuli, and defense against other organisms. Our findings revealed both genotype-specific and shared BP classes that were implicated in terminal drought tolerance (Supplementary Table S6).

### 3.4. The Potential Biological Function of Identified Proteins

To ascertain the possible biological function of DEPs identified in four genotypes under terminal drought stress, annotation was performed using the EuKaryotic Orthologous Groups (KOG) database. The upregulated and downregulated DEPs were classified into 25 groups based on KOG classes using *Arabidopsis* proteins as a reference. The top categories of enriched DEPs commonly found in both tolerant and sensitive genotypes for drought belonged to ribosomal structure and biogenesis, translation, posttranslational modifications (PTMs), protein turnover, chaperones, predicted function, carbohydrate and amino acid transport and metabolism (Supplementary Figure S2). Common KOG classes were identified in both tolerant and sensitive genotypes. These DEPs were grouped into several categories, including cellular processes and signaling, metabolism, information storage, and processing, as well as uncharacterized DEPs. Compared to sensitive genotypes, tolerant genotypes had fewer DEPs, with pyrophosphate-dependent phosphofructo-1-kinase being the only DEP in the metabolism category unique to the tolerant genotypes (Supplementary Table S7).

### 3.5. KEGG Pathways Enrichment

#### 3.5.1. Tolerant Genotypes

**Figure 3:**
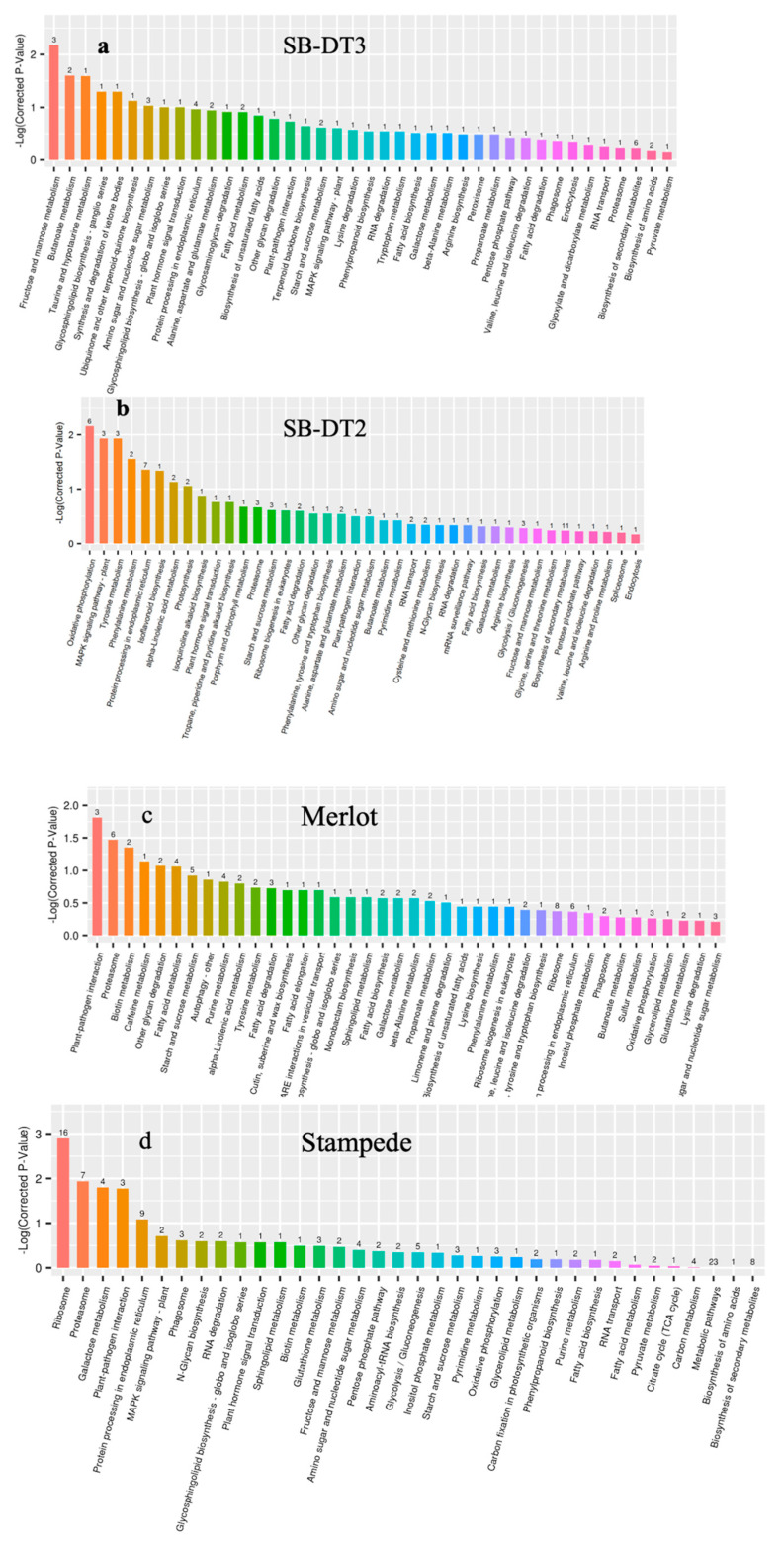
Significant enrichment of metabolic pathways in the four genotypes ((**a**) SB-DT3, (**b**) SB-DT2, (**c**) Merlot, (**d**) Stampede) in response to terminal drought stress.

To better understand the functional/GO classes assigned to identified DEPs, we utilized the KEGG database to map and categorize them into various metabolic pathways. The KEGG enrichment analysis revealed 44 metabolic pathways associated with DEPs in SB-DT3. These DEP-associated networks are further categorized into five groups: (1) cellular, (2) metabolism, (3) environmental, (4) genetic information processing, and (5) organismal systems (Supplementary Figure S3). The pathway enrichment analysis indicated that DEPs in SB-DT3 were predominantly involved in fructose, mannose, butanoate, taurine, and hypotaurine metabolisms, terpenoid-quinone, ubiquinone, and glycosphingolipidbiosyntheses, and synthesis and degradation of ketone bodies (Supplementary Figure S4). Notably, three pathways showed significant enrichment (*p* < 0.05): “fructose and mannose, butanoate, and taurine and hypotaurine metabolisms” (Figure 3).

**Figure 4:**
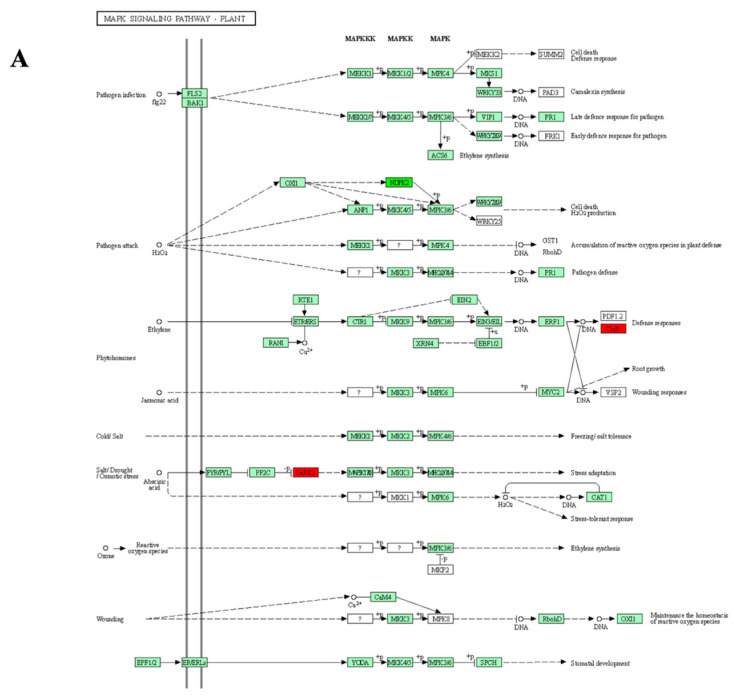

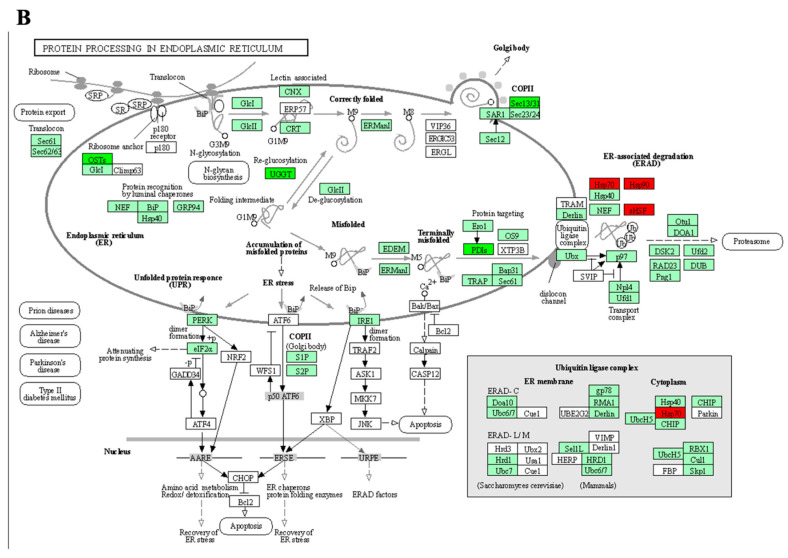
Pathway analysis of DEPs in response to terminal drought stress in tolerant genotype SB-DT2. (**A**) MAPK signaling and (**B**) protein processing in the endoplasmic reticulum pathways is shown. Proteins that were significantly upregulated in SB-DT2 under terminal drought stress are highlighted in red boxes, while proteins that did not show significant differential expression are indicated with a green background. The analysis reveals upregulation of specific proteins involved in MAPK signaling and protein processing pathways in the drought-tolerant SB-DT2 genotype.

In SB-DT2, we identified 48 metabolic pathways associated with DEPs. The enrichment analysis revealed the involvement of DEPs in several pathways, including oxidative phosphorylation, mitogen-activated protein kinase (MAPK) signaling, protein processing in endoplasmic reticulum, plant hormones signal transduction, tyrosine metabolisms, phenylalanine metabolisms, isoflavonoids biosynthesis, isoquinoline alkaloid biosynthesis, tropane, piperidine, and pyridine alkaloid biosynthesis. We identified six significant (*p* < 0.05) pathways in the tolerant genotype SB-DT2. More pathways were significantly enriched in SB-DT2 compared to SB-DT3 (Table 2) (Figure 4).

#### 3.5.2. Sensitive Genotypes

**Figure 5:**
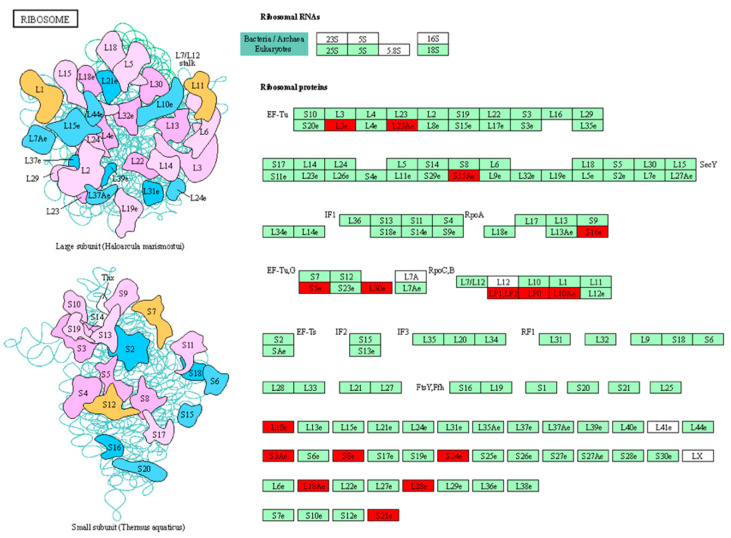
Ribosome pathway with the most upregulated DEPs identified in the sensitive genotype, Stampede under terminal drought stress.

In Merlot, 57 pathways were associated with enriched DEPs, and the significant (*p* < 0.05) pathways identified include plant–pathogen interaction, autophagy, biotin, and caffeine metabolisms. Similarly, in Stampede, 36 pathways were associated with enriched DEPs, and among these significant (*p* < 0.05) pathways identified include plant–pathogen interaction, ribosome, proteasome, and galactose metabolism. Merlot and Stampede demonstrated specific enriched pathways related to DEPs, with some commonalities such as plant–pathogen interaction and the proteasome. However, some differences in the pathways they exhibit were based on the enrichment factor and the number of enriched DEPs (Figure 5).

The pathway analysis revealed the key biological pathways altered in response to terminal drought stress that likely contributes to the drought tolerance mechanisms between the genotypes.

### 3.6. Corresponding Genes Expression in the Tolerant and Sensitive Genotypes

The results obtained from quantitative real-time PCR (qRT-PCR) revealed that the expression profiles of most of the genes in stressed and control samples corresponded to the expression patterns of DEPs. Genes associated with the tolerant genotypes SB-DT3 and SB-DT2, as well as the sensitive genotype Merlot, Stampede, were selected based on the upregulated DEPs (Supplementary Figure S5).

## 4. Discussion

Seed is the seminal material that determines the fate of a plant. The storage protein and moisture contents, germination rate, dormancy, and viability of a seed are important for its establishment as a seedling. However, the total protein content in the seed varies between diverse genotypes, species, or organisms. In addition, the variation in protein content between stress-tolerant and sensitive cultivars is due to spatial and temporal differences in gene expression that explicitly trigger specific pathways. Moreover, transcriptomes and associated proteomes of contrasting genotypes vary significantly due to environmental factors or when stress is induced by anthropogenic factors. Recently, with the availability of gel/label-free approaches, the proteomes of several stressed and unstressed legume species have been analyzed to identify significant DEPs and their associated pathways [35].

Similarly, we included two tolerant and sensitive genotypes and induced terminal drought stress after flowering to explore the variations in protein abundances between the seeds of these diverse genotypes. Understanding these genotype-specific protein changes can potentially aid in selecting resilient crop varieties and cultivating them to ensure better yields even under adverse environmental conditions.

### 4.1. Terminal Drought Stress and Abundance of Proteins

A higher number of up and downregulated proteins were identified in the sensitive genotypes compared to the tolerant ones. This discrepancy can be correlated to the difference in plant responses to drought stress, which varies with the genotype and stress severity and duration [36,37]. Additionally, the common proteins were higher in sensitive genotypes when compared with tolerant lines, potentially explaining the distinct molecular patterns associated with physiological traits of specific genotypes as reported in the common bean [36].

The HATPase_c domain-containing protein and X8 domain-containing protein showed significant differential abundances in SB-DT2 and SB-DT3 genotypes with a fold change of >2. The HATPase_c domain-containing protein identified here is a member of the histidine kinase family that has a conferred functional role in drought stress in *Arabidopsis* [38]. In another study, an examination of the molecular aspects of drought stress in Castanopsis fissa (chinquapin) plants uncovered the identification of proteins that carry the HATPase_c domain and are associated with the protein kinase B (kt) signaling pathway [39], whereas the X8 domain-containing protein identified here is similar to that of *Arabidopsis* proteins that act as a nonenzymatic ancillary domain responsible for callose binding [40]. Callose deposition, binding, and turnover proteins in *Arabidopsis* have been reported in response to environmental cues [41].

In contrast, sensitive genotypes showed a differential abundance of the 60S acidic ribosomal protein Po under terminal drought stress. In root proteomics analysis of tolerant grapevine genotypes, there was a fold change increase in the abundance of 60S acidic ribosomal protein Po compared to the control [42]. These findings suggest that the abundance of specific proteins varies between tolerant and sensitive genotypes, indicating distinct functional roles of these proteins in each genotype while combating terminal drought stress.

### 4.2. Heat Shock Proteins

In cellular homeostasis across diverse growth conditions, heat shock proteins (HSPs) emerge as integral molecular chaperones. Their pivotal role extends to ensuring proper protein folding during cellular processes, enhancing stability in membrane proteins, and orchestrating protein refolding in response to stress-induced perturbations. Notably, a diverse group of HSPs demonstrated chaperone function implicated in complex three-dimensional protein folding as a counteracting mechanism to the deleterious effects induced by stressors [43].

The significance of heat shock protein families like HSP60, HSP70, and HSP90 in plant abiotic stress responses has been underscored in prior studies [44]. The current investigation identified a few stress-responsive HSPs among four genotypes that include 14-3-3, small HSP, Clp R, and histidine kinase-like ATPases (HATPase_c) domain-containing proteins, and disulfide-isomerase. An increase in the stress protein, 14-3-3 that exhibits chaperone traits such as preventing aggregation and disentangling stress-denatured proteins has been reported in heat-tolerant pepper seedlings [45], and a later potential contributing role of 14-3-3 in heat tolerance has been corroborated [46]. Similarly, the HSPs identified in this study may have a potential role in drought stress tolerance.

### 4.3. Signal Transduction Mechanisms and Adaptive Responses during Stress

Under stress conditions, the signal transduction process commences with signal perception, followed by a cascade of phosphorylation events aimed at triggering stress-responsive genes. Simultaneously, this signal perception can also prompt the release of plant hormones, acting as regulatory molecules, thus perpetuating the signaling process [47]. Our examination of diverse genotypes revealed proteins integral to these signal transduction mechanisms. Specifically, the proteins identified as ethylene receptors in SB-DT3, protein kinase domain-containing protein in SB-DT2, serine/threonine-protein phosphatase in Merlot, and ethylene receptor in Stampede, all play roles within these signal transduction mechanisms [48]. These proteins suggest the role of signal transduction as one of the key mechanisms to facilitate adaptive responses in plants.

### 4.4. Protein Alterations in Plant Energy Balance for Drought Stress Resilience

The modulation of proteins involved in plant energy homeostasis could be a critical adaptive response to drought stress in plants. The imperative need for energy requirements under drought stress and elevated levels of ATP during energy homeostasis have been reported [49,50]. Intriguingly, a distinct divergence was observed in the abundance of production and energy conversion (EPEC) proteins between genotypes SB-DT3 and SB-DT2. The SB-DT3 exhibited a relatively lower proportion of EPEC proteins, whereas the SB-DT2 demonstrated a higher percentage. The EPEC proteins commonly identified in both tolerant genotypes include ATP synthase subunit alpha (chloroplastic), NADH dehydrogenase (ubiquinone) flavoprotein 1 (mitochondrial), ADP/ATP translocase, and PHB domain-containing protein. Similarly, the unique EPEC proteins identified in Merlot were malic enzyme, epimerase domain-containing protein, and ATP citrate synthase. The uniquely identified EPEC proteins in Stampede belonged to the vacuolar proton pump (V-ATPase) subunit B group. Notably, a pivotal role of the ATP1 subunit in wheat’s seed viability and development, particularly under ecologically challenging conditions characterized by drought, has been elucidated [51]. In such circumstances, the ATP1 subunit emerges as a key player, steadfastly upholding the continuous energy supply to plant cells, thereby serving as an indispensable determinant of their sustained survival. Similarly, diverse molecular variants of the NAD malic enzyme have been documented in the drought-stressed C4 plant, *Amaranthus* [52] These findings collectively underscore the intricate orchestration of protein-level adaptations in plants confronting drought stress, emphasizing the multifaceted strategies employed to ensure energy sufficiency and cellular survival.

### 4.5. Genotype Dependent Enrichment of Drought Stress Pathways

Our detailed analysis of DEPs in SB-DT3 showed the enrichment of three pathways. Previous studies have demonstrated the different roles of sugars as compatible solutes and signaling molecules under drought stress [53,54]. Relatedly, drought tolerance in wheat was linked to increased sucrose and mannose contents, as they observed higher amounts of these sugars in tolerant genotypes when compared with sensitive lines [55]. Moreover, pathways involving butanoate, taurine, and hypotaurine metabolisms were associated with drought responses in Loblolly pine (*Pinus taeda* L.) seedlings [56]. Additionally, compared to other genotypes, SB-DT2 exhibited more significantly enriched metabolic pathways associated with DEPs. As evidenced in this study, the role of the highly conserved MAPK signaling pathway in stress responses is suggested by regulating signal transduction and gene expression through MAPK cascades [57].

Interestingly, proteasome and plant–pathogen interaction pathways were enriched in both sensitive genotypes, suggesting that the dynamic changes in expressed proteins enable plant adaptation to environmental stresses. Indeed, combined transcriptomic and translational analyses of drought-stressed maize showed the importance of ribosome profiling in understanding stress responses [58]. We found extensive up and down-regulation of ribosomal proteins in the Stampede. This indicates an active stress response aimed at producing proteins to help the plant cope with water deficit conditions. Collectively, these findings provide insights into the existence of complex genotype-specific mechanisms in response to drought stress in plants.

## 5. Conclusions

In conclusion, our proteomic profiling revealed genotype-specific differences in protein abundance changes induced by terminal drought stress. The tolerant genotypes SB-DT3 and SB-DT2 exhibited fewer common drought-responsive proteins compared to the sensitive genotypes Merlot and Stampede, suggesting more dramatic proteomic perturbations are evident in drought-sensitive genotypes when compared with drought-tolerant ones, which is in concurrence with previous findings [52], while some biological processes like starch biosynthesis were commonly enriched across genotypes. However, our pathway analysis uncovered distinct genotype-specific responses. For instance, SB-DT3 displayed enrichment in metal ion and amino acid metabolism; MAPK signaling and secondary metabolism were enriched in SB-DT2; Merlot showed enrichment in pathogen defense, proteolysis, and biotin metabolism; and ribosomal function and galactose metabolism were enriched in Stampede. The sensitive varieties exhibited diverse biological processes, including macromolecule catabolism, energy reserve metabolism, responses to oxygen compounds, and biotic stresses. The genotype-specific proteomic and pathway signatures identified here may provide potential targets for molecular breeding efforts to enhance drought resilience in common beans. By elucidating proteomic networks associated with drought susceptibility versus tolerance, this work may assist in breeding genotypes with consistent yield in the field and genetic engineering to improve drought adaptation in common beans.

## Figures and Tables

**Table 1:** Shared DEPs identified in the tolerant genotypes and sensitive genotypes with fold change.

Proteins	Protein ID	SB-DT3 (FC)	SB-DT2 (FC)
Glycosyltransferase (EC 2.4.1.-)	V5N8Q0	0.66549411	0.504210124
Protein disulfide-isomerase (EC 5.3.4.1)	V7AXU4	0.389970224	0.265331957
MPN domain-containing protein	V7B979	0	0.246595377
HATPase_c domain-containing protein	V7C4I2	2.695036446	2.224064578
X8 domain-containing protein	V7C5Q7	4.00817599	0
ATP-dependent 6-phosphofructokinase (ATP-PFK) (Phosphofructokinase) (EC 2.7.1.11) (Phosphohexokinase)	V7CAY4	0.642129979	0.771054996
WD_REPEATS_REGION domain-containing protein	V7CUC0	1.415665913	1.60895747
**Proteins**	**Protein ID**	**Merlot (FC)**	**Stampede (FC)**
Alpha amylase inhibitor-1	A0T2V3	1.499790976	1.398607793
Thioredoxin-dependent peroxiredoxin (EC 1.11.1.24)	Q9FE12	0.512757684	0.662087976
1,4-alpha-glucan branching enzyme (EC 2.4.1.18)	Q9XIS5	0.38915774	0.731928462
60S acidic ribosomal protein Po	V7C7B8	2.252100651	3.055876326
Proteasome subunit beta	T2DN03	0.660628218	0.684574202
Proteasome subunit alpha type	V7AHS3	0.691908505	0.71020376
NADH dehydrogenase (ubiquinone) flavoprotein 1, mitochondrial (EC 7.1.1.2)	V7BMQ3	0.62391639	0.597474222
Alpha-galactosidase (EC 3.2.1.22) (Melibiase)	V7B2C4	0.757427659	0.755447862
Epimerase domain-containing protein	V7BF01	0.685465562	0.648430837
Mitochondrial Rho GTPase (EC 3.6.5.-)	V7BIE7	1.397530527	1.285438844
CYTOSOL_AP domain-containing protein	V7AYA6	0.699524201	0.726445174

**Table 2:** Metabolic pathways exhibiting significance (*p* < 0.05) are listed in the table for each genotype.

Genotypes	Pathways (*p* < 0.05)
SB-DT3	Fructose and mannose metabolism Butanoate metabolism Taurine and hypotaurine metabolism
SB-DT2	Oxidative phosphorylation MAPK signaling pathway—plant Tyrosine metabolism Phenylalanine metabolism Protein processing in endoplasmic reticulum Isoflavonoid biosynthesis
Merlot	Plant–pathogen interaction Proteasome Biotin metabolism
Stampede	Ribosome Proteasome Galactose metabolism Plant–pathogen interaction

## Data Availability

Data is contained within the article or Supplementary Material.

## Supplementary Materials

The following supporting information can be downloaded at https://www.mdpi.com/article/10.3390/biom14010109/s1. Supplementary Figure S1: overview of the proteomic data analysis of Tolerant and Sensitive genotypes. Supplementary Figure S2: KOG annotation of DEPs in (A) SB-DT3, (B) SB-DT2, (C) Merlot, and (D) Stampede. Supplementary Figure S3: DEPs annotated in the KEGG database in response to terminal drought stress are categorized into five main classes—Metabolism, Cellular Processes, Genetic Information Processing, Environmental Information Processing, and Organismal Systems. Supplementary Figure S4: top 20 enriched KEGG pathways for DEPs under terminal drought stress. The *x*-axis shows the top 20 pathways ranked by enrichment factor. The *y*-axis shows −log10(*p*-value). This plot highlights the key biological pathways altered in tolerant and sensitive genotypes in response to terminal drought stress. Supplementary Figure S5: expression patterns of coding genes corresponding to DEPs in tolerant and sensitive genotypes under stress and control conditions. Data are presented as mean (±SE). Supplementary Table S1: (a) common bean genotypes and their market class, (b) accumulated precipitation and temperature throughout the experiment, (c) various physiological parameters measured in four genotypes grown under terminal drought stress and control conditions. Supplementary Table S2: this table provides an overview of the various software tools utilized in the analysis pipeline of the current study, along with references and links for each tool. Supplementary Table S3: the table lists the DEPs along with the primer sequences for their corresponding coding genes. Supplementary Table S4: up and downregulated DEPs identified in the four genotypes. Supplementary Table S5: top 5 enriched GO terms for differentially expressed proteins, categorized by biological process, cellular component, and molecular function. Commonalities among genotypes highlighted. Supplementary Table S6: genotype specific and shared biological process in response to terminal drought stress in each genotype. Supplementary Table S7: KOG classification analysis of DEPs revealed proteins that were unique to, as well as shared between, the drought-tolerant and drought-sensitive genotypes.
