# Supplementary material for: Comprehensive Proteomic Analysis of Common Bean (Phaseolus vulgaris L.) Seeds Reveal Shared and Unique Proteins Involved in Terminal Drought Stress Response in Tolerant and Sensitive Genotypes
Source: Biomolecules. 2024 Jan 15;14(1):109. doi: 10.3390/biom14010109 (PMC10813106; doi:10.3390/biom14010109)
Supplement: Supplementary file 1 [file biomolecules-14-00109-s001.zip › Legend/Legends.pdf]

Legends

Supplementary Figure S1
Overview of the proteomic data analysis of Tolerant and Sensitive genotypes

Supplementary Figure S2
KOG annotation of DEPs in (A) SB-DT3 (B) SB-DT2 (C) Merlot (D) Stampede

Supplementary Figure S3
DEPs annotated in the KEGG database in response to terminal drought stress are categorized into five main classes: Metabolism, Cellular Processes, Genetic Information Processing, Environmental Information Processing, and Organismal Systems

Supplementary Figure S4
Top 20 enriched KEGG pathways for DEPs under terminal drought stress. The x-axis shows the top 20 pathways ranked by enrichment factor. The y-axis shows -log10(p-value). This plot highlights the key biological pathways altered in tolerant and sensitive genotypes in response to terminal drought stress.

Supplementary Figure S5
Expression patterns of coding genes corresponding to DEPs in tolerant and sensitive genotypes under stress and control conditions. Data are presented as mean (±SE).

Supplementary Table S1

a. Common bean genotypes and its market class
b. Accumulated Precipitation and Temperature Throughout the Experiment
c. Various physiological parameters measured in four genotypes grown under terminal drought stress and control conditions.

Supplementary Table S2
This table provides an overview of the various software tools utilized in the analysis pipeline of the current study, along with references and links for each tool.

Supplementary Table S3
The table lists the DEPs along with the primer sequences for their corresponding coding genes.

Supplementary Table S4
Up and down regulated DEPs identified in the four genotypes.

Supplementary Table S5
Top 5 enriched GO terms for differentially expressed proteins, categorized by biological process, cellular component, and molecular function. Commonalities among genotypes highlighted.

Supplementary Table S6
Genotype specific and shared biological process in response to terminal drought stress in each genotype.

Supplementary Table S7

KOG classification analysis of DEPs revealed proteins that were unique to, as well as shared between, the drought tolerant and drought sensitive genotypes.
