# Supplementary material for: Comprehensive Proteomic Analysis of Common Bean (Phaseolus vulgaris L.) Seeds Reveal Shared and Unique Proteins Involved in Terminal Drought Stress Response in Tolerant and Sensitive Genotypes
Source: Biomolecules. 2024 Jan 15;14(1):109. doi: 10.3390/biom14010109 (PMC10813106; doi:10.3390/biom14010109)
Supplement: Supplementary file 1 [file biomolecules-14-00109-s001.zip › Supplementary Tables/Supplementary Table S2.pdf]

Supplementary Table S2: This table provides an overview of the various software tools utilized in the analysis pipeline of the current study, along with references and links for each tool.

Tools	Description	Reference links
KOBAS 3.0	KEGG Orthology based annotation system	http://kobas.cbi.pku.edu.cn/
Blast2GO 2.8.3	Open-source platform for visualizing complex networks	http://www.cytoscape.org/
topGO. 2.26.0	R package for gene ontology enrichment analysis	https://bioconductor.org/packages/release/bioc/html/topGO.html
Database		
GO	Gene Ontology database	http://www.geneontology.org/
COG	Clusters of orthologous groups	http://www.ncbi.nlm.nih.gov/COG/
String	Functional protein association networks	http://string-db.org/
KEGG	The database of Kyoto Encyclopedia of Genes and Genomes	http://www.genome.jp/kegg/
