# Supplementary material for: Comprehensive Proteomic Analysis of Common Bean (Phaseolus vulgaris L.) Seeds Reveal Shared and Unique Proteins Involved in Terminal Drought Stress Response in Tolerant and Sensitive Genotypes
Source: Biomolecules. 2024 Jan 15;14(1):109. doi: 10.3390/biom14010109 (PMC10813106; doi:10.3390/biom14010109)
Supplement: Supplementary file 1 [file biomolecules-14-00109-s001.zip › Supplementary Tables/Supplementary Table S1.pdf]

Supplementary Table S1

a. **Common bean genotypes and its market class** (Urrea *et al* 2022)

	PEDIGREE	Market Class	ORIGIN
1	SB-DT2	Pinto	NE28-18-18-14
2	SB-DT3	Small red	NE28-18-18-21
3	Merlot	Small red	NE28-18-18-31
4	Stampede	Pinto	NE28-18-18-32

b. **Accumulated Precipitation and Temperature Throughout the Experiment**

Rain before/after stress	Irrigation before stress (Stress)	Irrigation before/after stress (Control)	Total water (irrigation + precipitation) (Stress)	Total water (irrigation + precipitation) (Control)	Temperature over 32.2 °C (no of times)	Temperature over 37.8 °C (no of times)
37.6 mm/ 8.6 mm (Total rainfall 46.2 mm)	101.6 mm	342.9 mm	147.8 mm	389.1 mm	45	1

c. **Various physiological parameters measured in four genotypes grown under terminal drought stress and control conditions.**

	Terminal Drought Stress				Control			
**PEDIGREE**	**Yield lbs/acre**	**Flowering days**	**Maturity days**	**100-SW gr**	**Yield lbs/acre**	**Flowering days**	**Maturity days**	**100-SW gr**
SB-DT3	1817	47	89	33.2	3482	48	97	32.6
SB-DT2	2490	45	87	34.2	3962	46	94	37.5
Merlot	2251	48	89	34.6	3241	47	99	37.1
Stampede	2416	49	87	34.6	3781	48	98	34.7

SW- Seed weight
