# Supplementary material for: Comprehensive Proteomic Analysis of Common Bean (Phaseolus vulgaris L.) Seeds Reveal Shared and Unique Proteins Involved in Terminal Drought Stress Response in Tolerant and Sensitive Genotypes
Source: Biomolecules. 2024 Jan 15;14(1):109. doi: 10.3390/biom14010109 (PMC10813106; doi:10.3390/biom14010109)
Supplement: Supplementary file 1 [file biomolecules-14-00109-s001.zip › Supplementary Tables/Supplementary Table S3.pdf]

Supplementary Table S3: The table lists the DEPs along with the primer sequences for their corresponding coding genes.

Protein ID	Protein Names	Primers
	**SB-DT3**	
V7BAM6	SHSP domain-containing protein	GTGCTTTAATCTGATCCACCT<br>CGGATGGGAATGTTCTACAC
V7ALP9	Peptidylprolyl isomerase	CTGGTTCTCCTCCAACTATTC<br>TCATCAAGATCCTTGGGATTC
V7AED4	PfkB domain-containing protein	GTCGTTCTCCTTGAGGATTG<br>CGAGATGCTCATCGACTTC
V7C5Q7	X8 domain-containing protein	GTCCTTCAAGTGAACCACTC<br>CACACCATCCACCTTAACTC
V7CH04	Acyl-[acyl-carrier-protein] desaturase (EC 1.14.19.-)	GAACTGAGAGAGAGAGCAAAG<br>AGACTGCCCAGGAAGTAA
	**SB-DT2**	
V7C6L3	PPDK_N domain-containing protein	CTTTGCGGTGAGTGTGAA<br>TCGGAAGCCGGATTAGT
V7APP2	40S ribosomal protein S8	CGCAAGACGCGTATACTT<br>GCTGTCTTCTTCTTCCTACC
V7CPQ1	AAA domain-containing protein	CTGACAAGACGTGACTCATC<br>GAAGAAACTATCTGGACCCATT
V7AM91	Ribosomal_L16 domain-containing protein	GATTAAGAACAAGCCCTACCC<br>CATTCTCCTTCTCCCAACTAAC
	**Merlot**	
V7C7B8	60S acidic ribosomal protein P0	CCACAAGCCCATATGAGAAG<br>GCTTAACATTCCGACCAAGA
V7B517	Sacchrp_dh_NADP domain-containing protein	CGGCTTCACAGGCAAATA<br>AGAGAGAGTGGAGGGTTG
V7CPR7	SHSP domain-containing protein	TCTCTTTCTTCCTCCTTCCC<br>CTACCTTCACTTCCTCCTTCT
V7BGT1	Eukaryotic translation initiation factor 3 subunit M (eIF3m)	AGCTCTCTCTGTTCTGGTATC<br>TTAGCCATTGATGGGAAAGTC
	**Internal Control**	
V7BQX5	Pv β-tubulin	CGTCCTCATGGATCTTGAAC<br>CTTCAGTGTAGTGGCCTTTG
