# Supplementary material for: Comprehensive Proteomic Analysis of Common Bean (Phaseolus vulgaris L.) Seeds Reveal Shared and Unique Proteins Involved in Terminal Drought Stress Response in Tolerant and Sensitive Genotypes
Source: Biomolecules. 2024 Jan 15;14(1):109. doi: 10.3390/biom14010109 (PMC10813106; doi:10.3390/biom14010109)
Supplement: Supplementary file 1 [file biomolecules-14-00109-s001.zip › Supplementary Tables/Supplementary Table S6.pdf]

Supplementary Table S6: Genotype specific and shared biological process in response to terminal drought stress in each genotype

Genotypes	Most significant Biological Process
SB-DT3	starch biosynthetic process<br>protein-chromophore linkage<br>photosynthesis, light reaction<br>response to metal ion<br>glutamine family amino acid metabolic pr...<br>defense response<br>response to cadmium ion<br>glutamine family amino acid biosynthetic...<br>glycogen metabolic process<br>glycogen biosynthetic process
SB-DT2	protein-chromophore linkage<br>photosynthesis, light reaction<br>starch biosynthetic process<br>response to abscisic acid<br>glycogen biosynthetic process<br>defense response<br>multi-organism process<br>response to biotic stimulus<br>electron transport chain<br>response to inorganic substance
Merlot	protein-chromophore linkage<br>photosynthesis, light reaction<br>starch biosynthetic process<br>macromolecule catabolic process<br>electron transport chain<br>defense response<br>glycogen metabolic process<br>glycogen biosynthetic process<br>energy reserve metabolic process<br>protein catabolic process
Stampede	protein-chromophore linkage<br>photosynthesis, light reaction<br>starch biosynthetic process<br>response to inorganic substance<br>response to oxygen-containing compound<br>defense response<br>response to water<br>response to acid chemical<br>defense response to other organism<br>multi-organism process
