# Supplementary figures and images for: Comprehensive Proteomic Analysis of Common Bean (Phaseolus vulgaris L.) Seeds Reveal Shared and Unique Proteins Involved in Terminal Drought Stress Response in Tolerant and Sensitive Genotypes
Source: Biomolecules. 2024 Jan 15;14(1):109. doi: 10.3390/biom14010109 (PMC10813106; doi:10.3390/biom14010109)

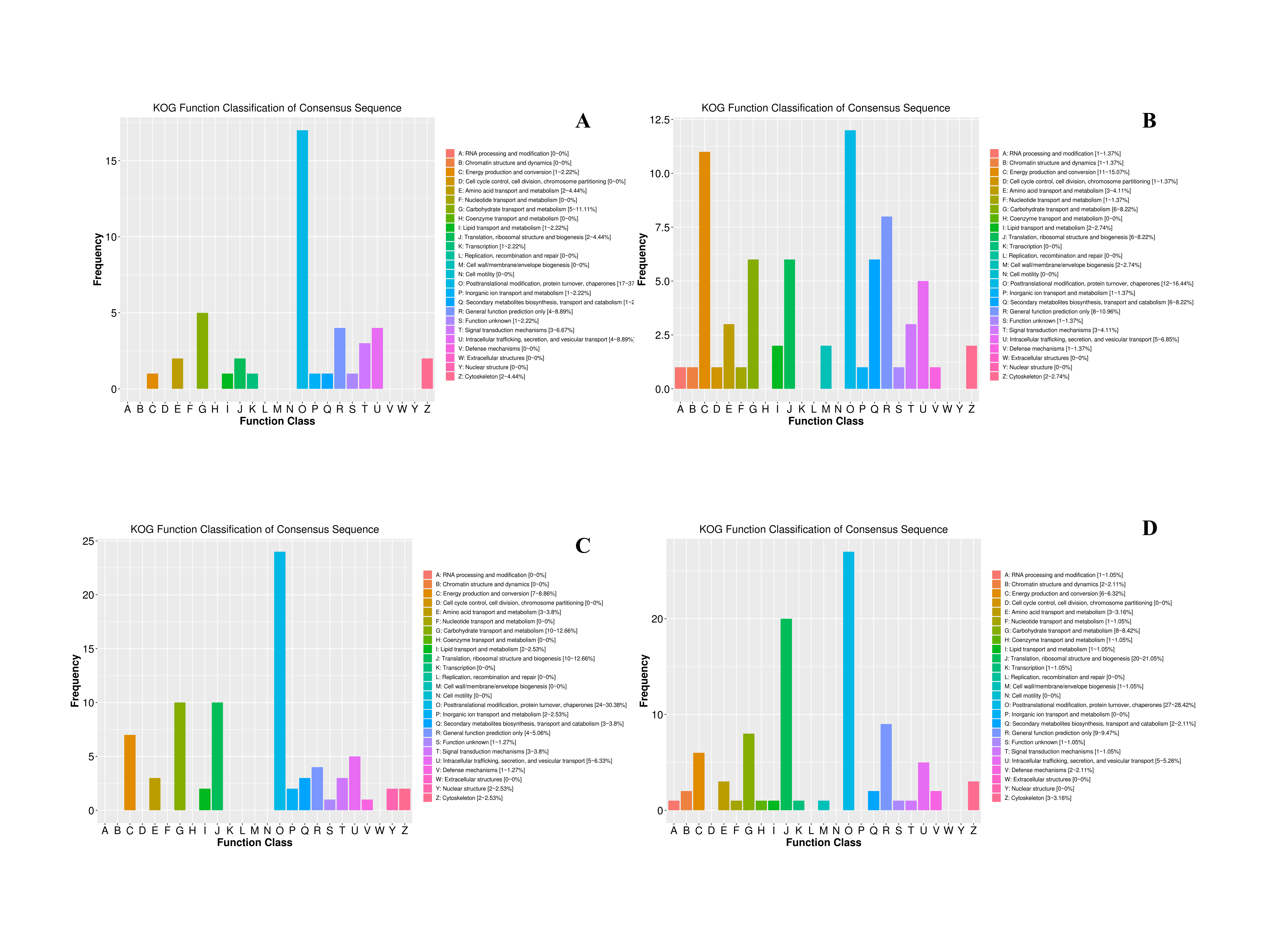

Supplement: Supplementary file 1 [file biomolecules-14-00109-s001.zip › Supplementary Figurs/Supplementary Figure S2.tif]

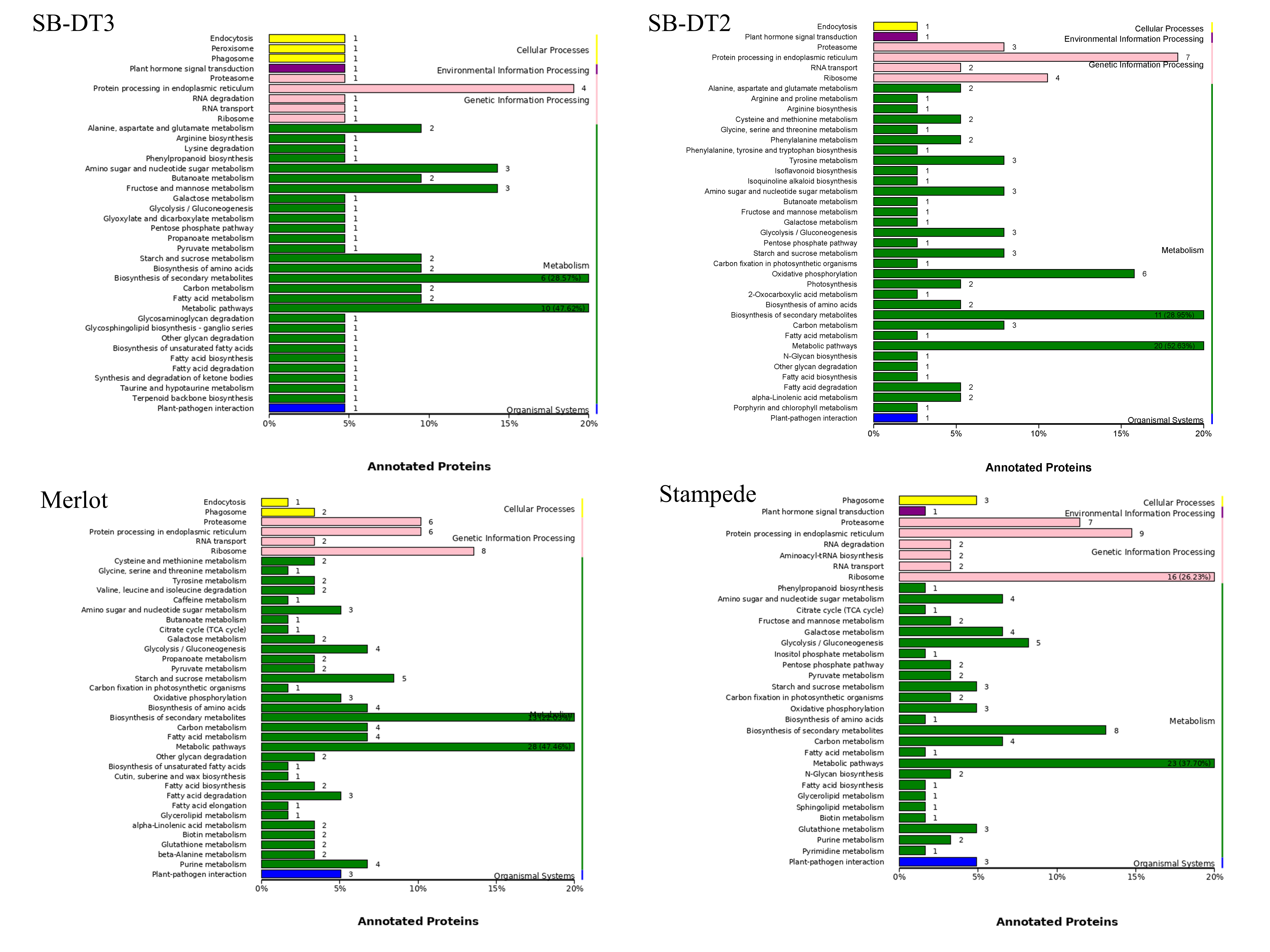

Supplement: Supplementary file 1 [file biomolecules-14-00109-s001.zip › Supplementary Figurs/Supplementary Figure S3.tif]

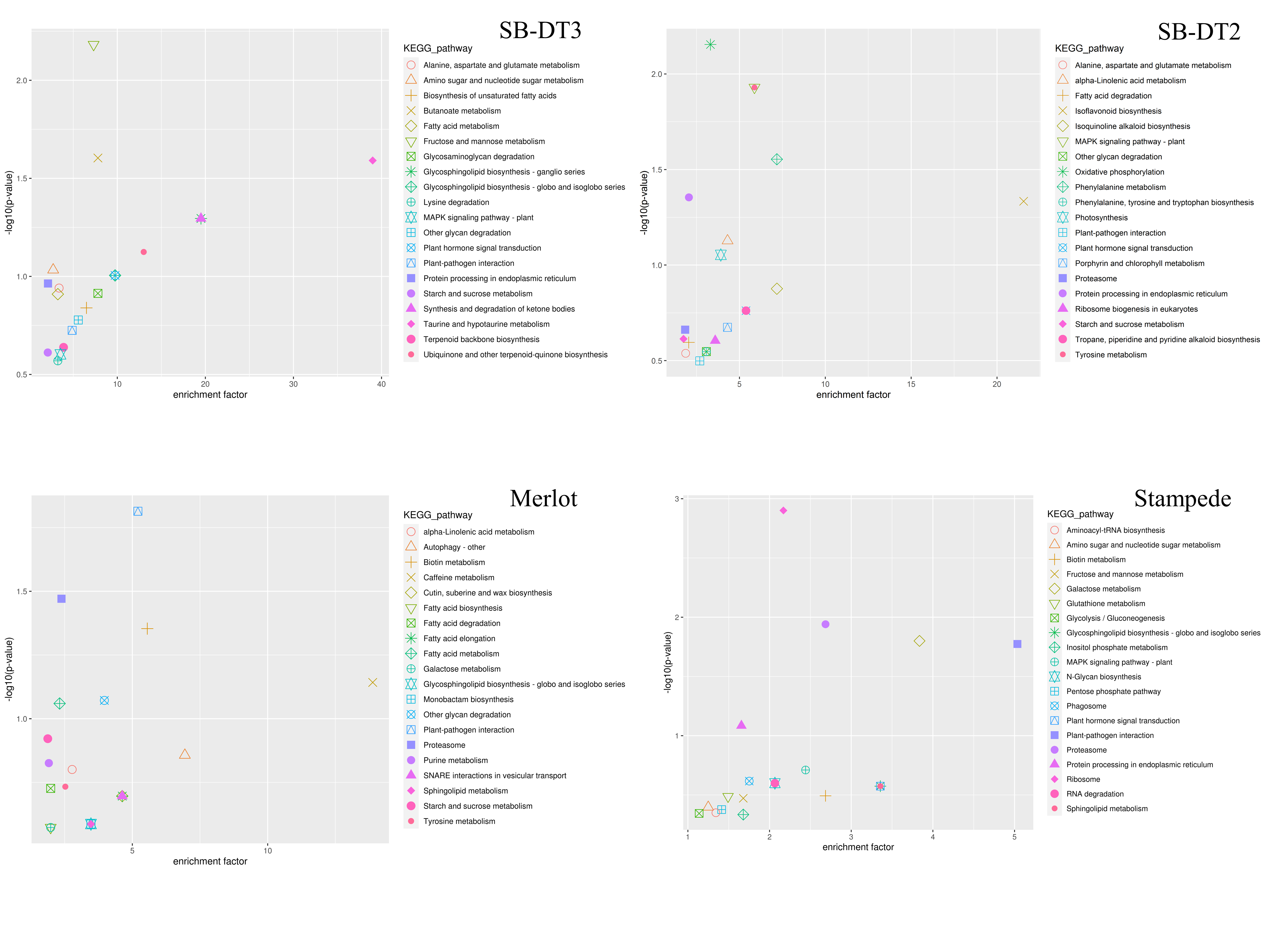

Supplement: Supplementary file 1 [file biomolecules-14-00109-s001.zip › Supplementary Figurs/Supplementary Figure S4.tif]

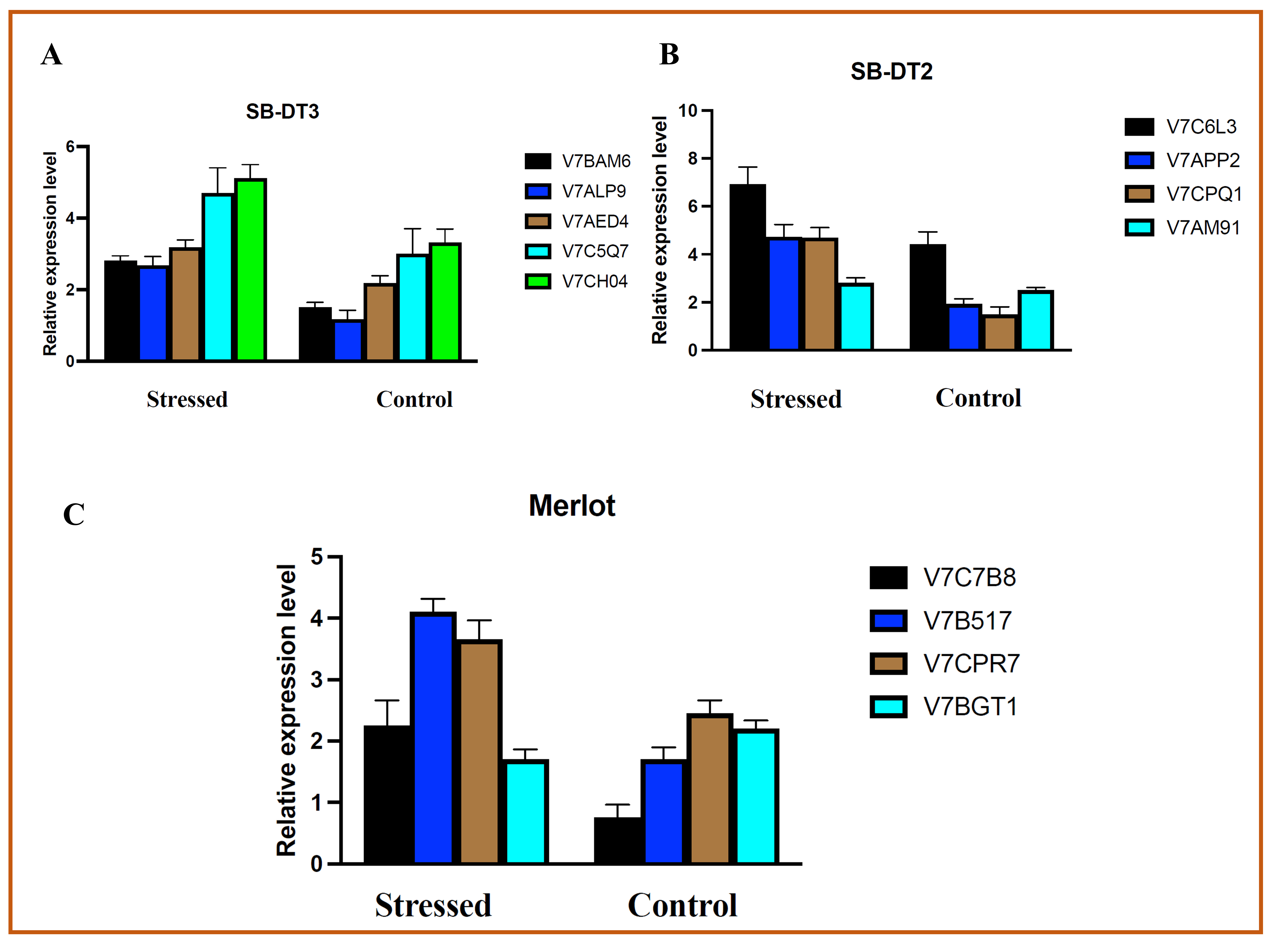

Supplement: Supplementary file 1 [file biomolecules-14-00109-s001.zip › Supplementary Figurs/Supplementary Figure S5 .tif]
